# Subject-Specific Finite Element Modelling of the Human Hand Complex: Muscle-Driven Simulations and Experimental Validation

**DOI:** 10.1007/s10439-019-02439-2

**Published:** 2019-12-16

**Authors:** Yuyang Wei, Zhenmin Zou, Guowu Wei, Lei Ren, Zhihui Qian

**Affiliations:** 1 Department of Mechanical, Aerospace and Civil Engineering, The University of Manchester, Manchester, UK; 2 Key Laboratory of Bionic Engineering, Ministry of Education, Jilin University, Changchun, China; 3 Institute of Mechanical Engineering, University of Salford, Manchester, UK

**Keywords:** Finite element human hand model, Finite element method, Electromyography, Biomechanics, Haptics

## Abstract

This paper aims to develop and validate a subject-specific framework for modelling the human hand. This was achieved by combining medical image-based finite element modelling, individualized muscle force and kinematic measurements. Firstly, a subject-specific human hand finite element (FE) model was developed. The geometries of the phalanges, carpal bones, wrist bones, ligaments, tendons, subcutaneous tissue and skin were all included. The material properties were derived from *in-vivo* and *in-vitro* experiment results available in the literature. The boundary and loading conditions were defined based on the kinematic data and muscle forces of a specific subject captured from the *in-vivo* grasping tests. The predicted contact pressure and contact area were in good agreement with the *in-vivo* test results of the same subject, with the relative errors for the contact pressures all being below 20%. Finally, sensitivity analysis was performed to investigate the effects of important modelling parameters on the predictions. The results showed that contact pressure and area were sensitive to the material properties and muscle forces. This FE human hand model can be used to make a detailed and quantitative evaluation into biomechanical and neurophysiological aspects of human hand contact during daily perception and manipulation. The findings can be applied to the design of the bionic hands or neuro-prosthetics in the future.

**Electronic supplementary material:**

The online version of this article (10.1007/s10439-019-02439-2) contains supplementary material, which is available to authorized users.

## Introduction

Hands are used to perceive and manipulate objects during daily life. The sense of touch and its coding mechanism make it possible for us to interact with this world. From the biomechanical point of view, our understanding of the biological function of the sophisticated bony structure and tendon topology remains primitive. In the study of motor control, the muscle synergy controlling strategies and their resultant grasping stability need to be well investigated before its application to the bionic or robotic hand design. Most importantly, in haptic or neurophysiological investigation, afferent tactile signals cannot be well-captured during active touch or manipulation procedure. When an object is sensed or manipulated, neural impulse signals are generated at the mechanoreceptors located underneath the epidermis according to the shape and texture of the object.^22^,^43^,^47^ These mechanoreceptors receive and encode the mechanical information such as strain or strain energy density (SED) and send the encoded information to somatosensory cortex.^14^,^38^ However, these mechanical parameters such as the SED are not measurable and observable with current technology. There is no practical method to investigate the neurophysiological aspects of hand perception during manipulation.^23^,^44^,^45^ Therefore, a digital or mathematical model of a human hand is urgently needed for the ergonomic, biomechanical and neurophysiological investigations of tactile mechanics. It will be a powerful numerical tool for the design of a bionic or haptic interfaced hand.

During the last decade, researchers started to focus on the contact mechanism of the human hand or finger pad using simplified two dimensional FE models.^18^,^41^ Although 2D FE models can be used to predict the contact, it has been found that the geometry of the model is critical for simulating hand contact mechanisms and mechanoreceptor responses.^10^ The muscle forces cannot be included in these 2D FE human finger models since the tendon insertion points cannot be created on the two-dimensional structure; therefore, the whole sensorimotor control loop cannot be formed.^18^ Recently, some 3D finite element models have been developed to gain a more realistic simulation of human hand contact. Dandekar *et al*.^10^ created 3D FE models of human and monkey finger pads with multi-layered structures to study the response of slowly adapting type I mechanoreceptor to different stimuli. The strain energy density was found to be a good candidate to quantify the stimulus received by the Merkel corpuscle. However, only the geometry of a fingertip was developed, so muscle force cannot be applied and the active touch process cannot be simulated. Chamoret *et al*.^7^ created a whole hand FE model which contained bones, soft tissues and skin reconstructed from CT images. The material properties of skin and subcutaneous tissues were simplified as isotropic linear elastic. Cylindrical grasping was simulated and the contact pressure map was calculated based on a customized contact algorithm. A similar FE hand model was developed by Chamoret *et al*.^8^ with anisotropic hyperelastic material properties, but only a simple contact between the fingertip and a rigid plate was simulated with the fingers being fully extended. Both FE models were not well-validated, because the geometry of the finite element hand model was reconstructed from one subject, but the grasping test for validation was performed using a different subject. The contact area was not included in the validation. Their FE models did not consider the subject-specific loading conditions or muscle forces neither. They are therefore not suitable for investigating sensorimotor control strategies and the neurophysiological phenomenon. Another simplified FE hand model was developed by Harith^19^ to study the contact mechanism of the human hand. The geometry of the bones and tissues were considered and isotropic hyperelastic rubber-like material behaviour was assigned to the soft tissues. This FE hand model was also partly validated by comparing the predicted contact area with the *in-vivo* test results. However, the two-layered structure of the skin was not modelled in Harith’s FE hand, so the predicted contact related results were not comparable with real-world data.

To the authors’ knowledge, there is no subject-specific FE hand model available in the literature that applied realistic geometry and reasonable material properties. Very few finite element analyses have considered the muscle forces to simulate the grasping or contact mechanisms of an anatomically intact human hand. None of the existing FE hand models have ever been validated. There is an urgent need to construct a comprehensive FE model for a better and accurate simulation of the human hand. This paper explains the process of developing a subject-specific FE human hand which contains the geometry of the wrist bones, carpal bones, phalanges, subcutaneous tissue and skin. The muscle forces and kinematic motion data were captured from the *in-vivo* grasping tests of the specific subject. Three main grasping postures used during our daily lives were then simulated: cylindrical grasping, spherical grasping and precision grasping. This FE model was finally validated by comparing the predicted contact pressure and contact area against *in-vivo* test results. Sensitivity analysis was performed to investigate the effects of variations in muscle forces and material properties on model predictions.

## Materials and Methods

### FE Model Construction

#### 3D Geometry Construction

**Figure 1:**
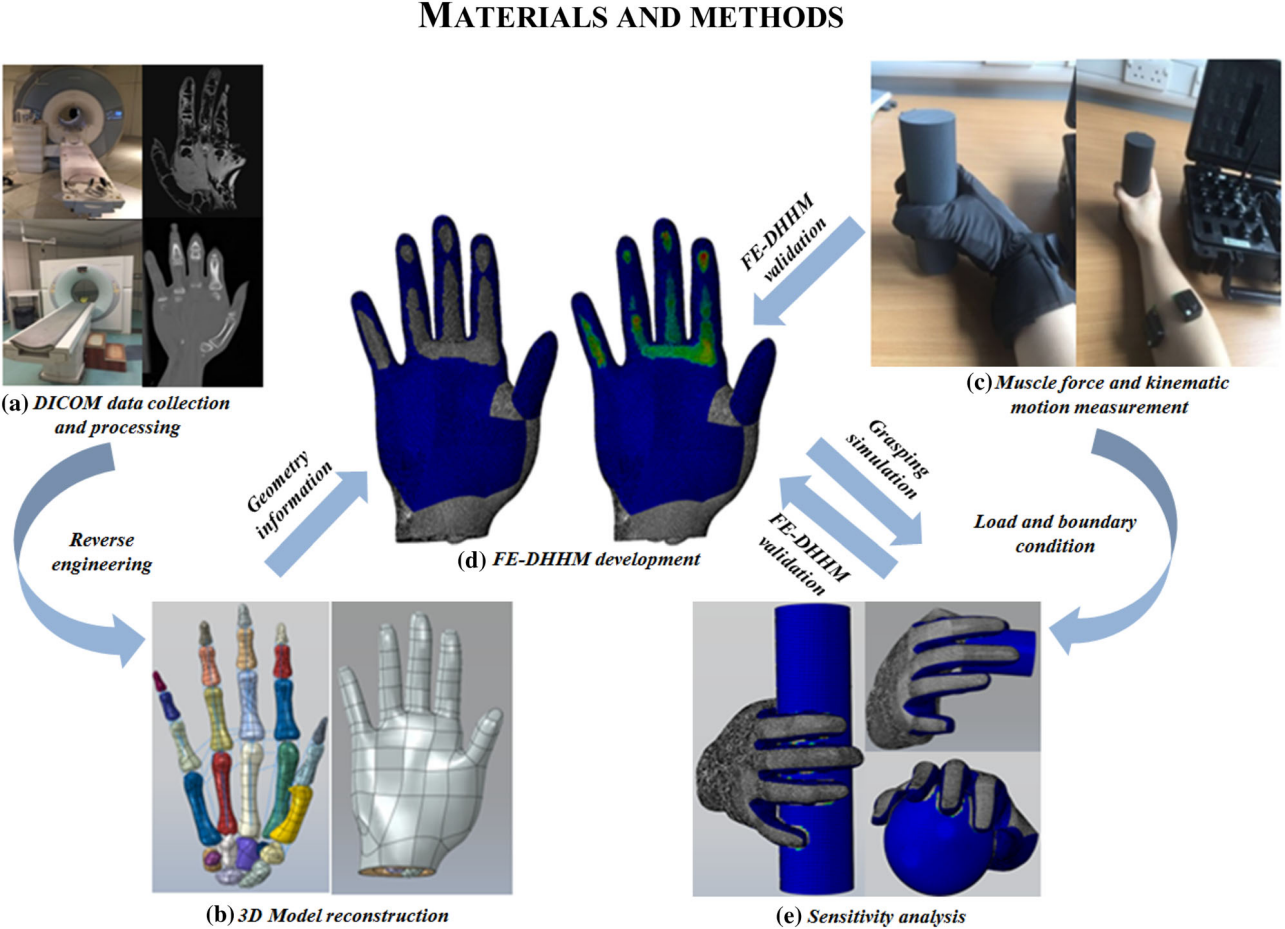
Main procedure of this study. (a) DICOM data collected from a specific subject. (b) 3D model reconstruction. (c) The kinematic motion data and muscle forces collected from the *in-vivo* experiments. (d) Predicted contact area and contact pressure. (e) The sensitivity analysis.

To obtain the geometrical information for a subject-specific FE human hand, CT and MR images were taken from a 23-year-old healthy male. These 2D data was processed using the medical image processing software Mimics (Materialise, Leuven, Belgium). All the CT images were segmented manually into the bones and skin, while the subcutaneous tissues and tendons were reconstructed based on MR images (see Fig. 1). The obtained geometry was then exported as STL mesh which was subsequently converted into solid models in Creo (PTC, Creo Parametric, US). The anatomical positions of the bones and soft tissues were aligned according to the MR images. Finally all the anatomical structures including the bones (14 phalangeal, 5 metacarpal, and 8 carpal bones), subcutaneous tissues and skin were imported into Abaqus (Simulia, Providence, US). Solid element C3D4H was used to mesh all parts of the FE human hand (see Fig. 1). The ligaments were modelled by spring elements to mimic the supporting tissues around the joints. The anatomical locations of the ligaments were determined according to the MR images.

#### Material Properties

Skin is a heterogeneous, extremely anisotropic, viscoelastic material and with complex behaviour. Many researchers have conducted *in-vivo* or *in-vitro* experiments such as uniaxial tensile and compression tests to find the constitutive equation which represents the behaviour of the skin.^16^,^35^,^36^ FE simulation has also been employed to conduct a parametric study to quantify the hyperelastic parameters of human skin.^9^,^31^,^33^ Existing investigations show that the Ogden model can provide the best fit of the hyperelastic behaviour of human skin.^2^,^31^ The Ogden model regards soft tissues as an incompressible material governed by the strain energy U as indicated in Eq. (1)

1 $$ U = \mathop \sum \limits_{i = 1}^{N} \frac{{2\mu_{i} }}{{\alpha_{i}^{2} }}\left( {\overline{\lambda }_{1}^{{\alpha_{i} }} + \overline{\lambda }_{2}^{{\alpha_{i} }} + \overline{\lambda }_{3}^{{\alpha_{i} }} - 3} \right) + \mathop \sum \limits_{i = 1}^{N} \frac{1}{{D_{i} }}\left( {J^{el} - 1} \right)^{2i} $$

where $\overline{\lambda }_{i}$ are the deviatoric principal stretches, *N* stands for the number of material parameter, $\mu_{i} ,  \alpha_{i}$ and $D_{i}$ are temperature-dependent material parameters. $J^{el}$ relates to the total volume ratio. The initial shear and bulk modulus are given in Eq. (2)

2 $$ \mu_{0} = \mathop \sum \limits_{i = 1}^{N} \mu_{i} ,\,K_{0} = \frac{2}{D} $$

The compressibility was defined by specifying a non-zero value for $D_{i}$ which is related to Poisson’s ratio $\nu$ as shown in Eq. (3)

3 $$ D_{i} = \frac{2}{{K_{0} }} = \frac{{3\left( {1 - 2\nu } \right)}}{{\mu_{0} \left( {1 + \nu } \right)}} $$

The material parameters for defining soft tissues using the Ogden model were extracted from other researchers’ uniaxial tensile tests on human skin and fat tissue specimens.^16^,^36^ This nominal stress-strain data was fitted into Eq. (1) by using the ‘material evaluate’ module in Abaqus. A similar method has been used by other researchers and the predicted results have shown a good agreement with the experiment results.^18^,^19^ The material parameters thus obtained for determining the hyper-elastic behaviour of soft tissues are shown in Tables 1 and 2.

**Table 1:** Material property of skin.

*i*	*μ*_*i*_ (MPa)	*α*_*i*_
1	− 0.07594	4.941
2	0.01138	6.425
3	0.06572	4.712

**Table 2:** Material property of subcutaneous tissue.

*i*	*μ*_*i*_ (MPa)	*α*_*i*_
1	− 0.04895	5.511
2	0.00989	6.751
3	0.03964	5.262

The bones were considered as isotropic linear elastic, with Young’s modulus of 17 GPa and Poisson ratio of 0.3.^7^,^19^ The stiffness of the ligaments/spring elements were obtained from the existing data available in the literature^6^,^29^,^46^ and the detailed information was presented in Table S1 in the supplementary material of this paper.

The contact setting is critical for this FE hand model because the contact algorithm has a significant influence on the simulated contact pressure and other mechanical parameters.^18^ Two key issues of contact definition in the FE model are the impenetrability and friction.^7^ The ‘hard contact’ was defined between the hand and the grasped object, allowing no penetration of the element nodes into another surface of the contact pair. Frictional surface-to-surface contact behaviour was defined to allow sliding between the skin and the objects with a friction coefficient of 0.74.^28^

#### In-Vivo Grasping Tests, Motion Measurement and Muscle Force Estimation

The same subject who undertook the CT/MR scan performed three different *in-vivo* grasping tests (cylindrical grasping, spherical grasping, precision grasping) which are the most frequently used postures during daily life.^24^,^30^ Three different objects were grasped and each grasping action was performed six times. The subject gave informed consent to participate in the MRI scanning and motion capture measurements, which were approved by the Ethics Committee of the First Hospital of Jilin University.

The kinematic motion data was captured using a VMG30 data glove (Virtual Motion Lab, Dallas, US) (see Fig. 1). The glove was equipped with bending sensors at the interphalangeal and metacarpophalangeal joints to capture the joint angles accurately. The contact pressures on fingertips were also recorded by the pressure sensors located on the finger pads. This allows the contact pressures and corresponding kinematic motion to be recorded simultaneously.

**Figure 2:**
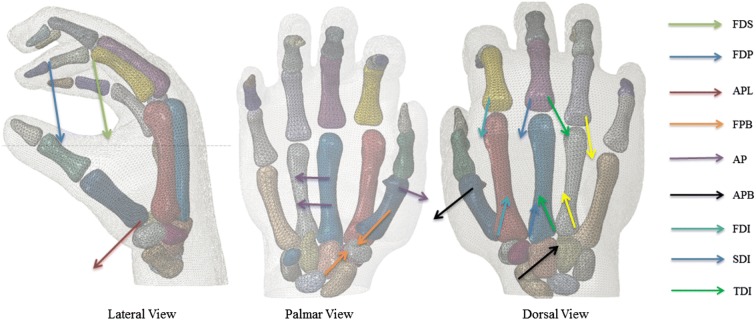
Muscle force definition in the FE human hand. The arrows represent directions and anatomical positions of the applied muscle forces for all three defined grasping (Spherical grasping is shown in this diagram). The full names of the muscles are given in the text below.

There are 14 extrinsic muscles in the human forearm and 18 intrinsic muscles located in hand which affect the motion of the hand. Among them, three extrinsic and six intrinsic muscles associated with hand grasping were selected for measuring the muscle forces.^32^,^40^ All of the nine muscle forces shown in Fig. 2 were estimated based on the electromyography (EMG) signals which were captured by Delsys wireless EMG system (Delsys Inc., Boston, US) (see Fig. 1) during the *in-vivo* grasping test. Each Trigno sensor was placed along muscle fibres according to the guidelines of the surface electromyography for the non-invasive assessment of muscles.^20^ Before the isometric grasping test, maximum voluntary contraction (MVC) tests^25^,^26^ were carried out for all nine muscles involved using Jamar dynamometer. The recorded EMG data were band-pass filtered (20–400 Hz) with a Butterworth filter and rectified. The muscle forces during grasping were then derived based on the maximum voluntary contraction forces and the assumption that for isometric muscle contracting, there would be a linear relationship between the EMG signal and muscle force.^5^,^27^,^37^ Similar method has been employed by other researchers to calculate muscle forces of isometric contraction.^5^,^11^,^12^

#### Loading and Boundary Conditions

In total, nine muscle forces were applied as concentrated loads onto the insertion points of the corresponding ligaments or tendons: flexor digitorum superficialis (FDS), flexor digitorum profundus (FDP), abductor pollicis longus (APL), flexor pollicis brevis (FPB), adductor pollicis muscle (AP), abductor pollicis brevis (APB) and three dorsal interossei muscles (FDI, SDI, TDI). The magnitudes of these muscle forces were listed in Table 3. All the anatomical positions where the forces were applied were determined based on MR images.

**Table 3:** Muscle forces during grasping for one of the six trails.

Muscle	Cylindrical grasping (N)	Spherical grasping (N)	Precision grasping (N)
FDS	138.78	240.03	111.72
FDP	89.37	155.84	162.20
APL	32.01	14.87	51.07
APB	6.74	6.04	6.11
AP	26.68	11.10	115.36
FPB	105.23	57.00	102.60
FDI	41.40	46.01	42.17
SDI	68.11	64.50	61.70
TDI	41.20	39.49	32.62

The kinematics of the hand depends mainly on the motions of the skeleton and the soft tissues. The supporting structures around each joint such as ligaments and tendons are also critical to the bio-mechanical movements. In this research, kinematics was defined to simulate the rotations at the finger joints.

**Figure 3:**
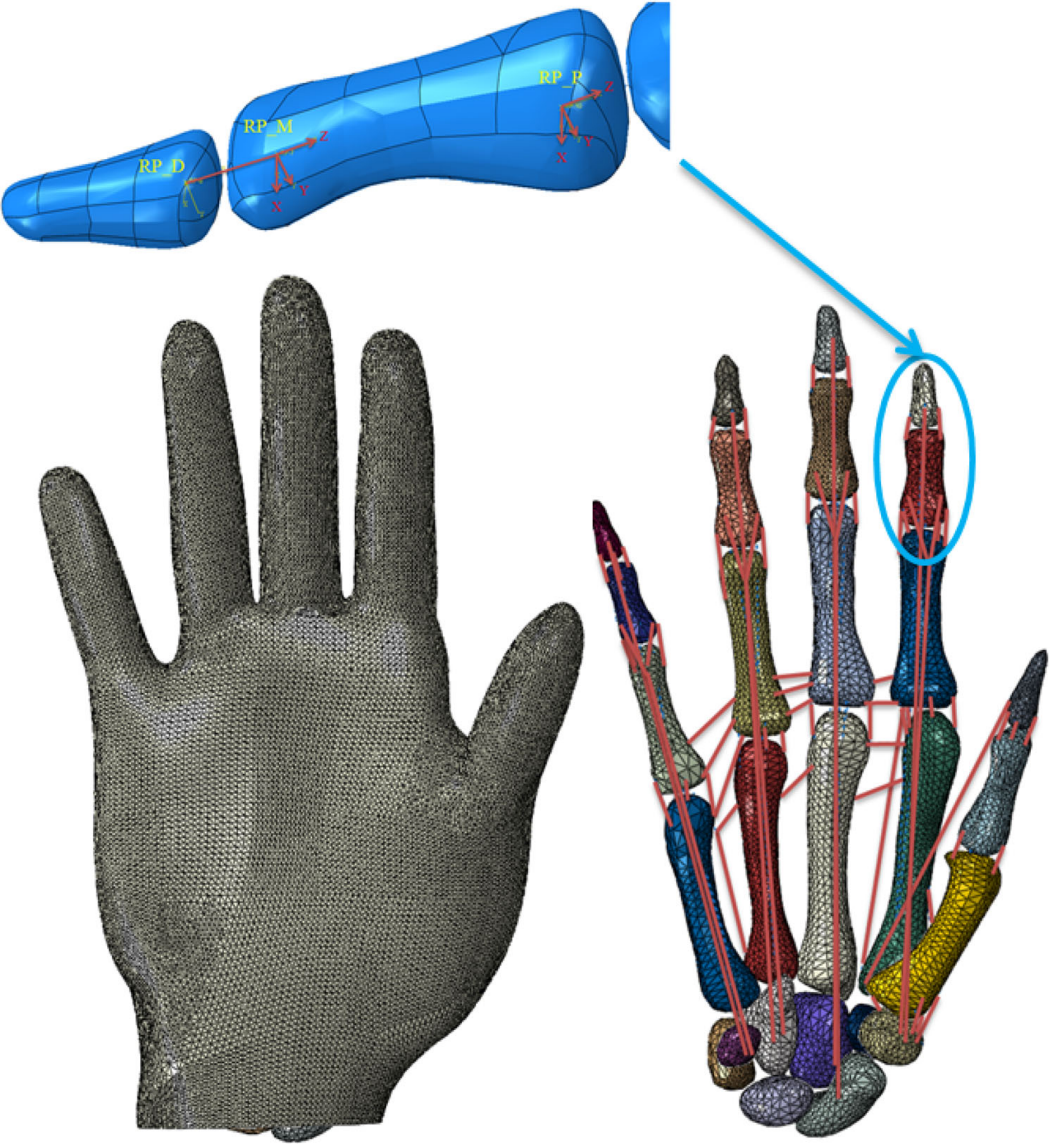
Kinematic motion definition and anatomical view of the FE hand model. The red lines represent ligaments and tendons. The diagram on the top shows the boundary conditions defined at each interphalangeal joint.

For the joint between the distal and middle phalanges shown in Fig. 3, two reference points were created; point RP_D at the proximal head of the distal phalanx and point RP_M at the rotational centre of the distal phalanx. Point RP_D was then kinematically related to the distal bone using constraints. A local co-ordinate system was generated with its origin at point RP_M. Its ‘*X*’ axis was along the rotational axis of the joint, the ‘*Z*’ axis along the longitudinal direction of the middle phalanx. A rigid wire was created to define a connector between points RP_D and RP_M. This connector only allowed point RP_D to rotate around the ‘*X*’ axis. A third reference point, RP_P, was also specified at the proximal head of the medial phalanx and is related to medial bone and reference point RP_M, so that the distal phalanx also had the ability to rotate with the medial phalanx.

The kinematics of the other joints was defined in a similar way as illustrated above. The only difference is that for the metacarpal joint, the rigid wire was also allowed to rotate along the ‘*Y*’ axis to mimic the adduction/abduction of the MCP joints. This fully developed FE human hand has 27-degree-freedom assigned onto the 15 interphalangeal and the wrist joints. Angular displacements were finally specified at each joint according to the measured angles to define a biomechanically realistic and numerically stable skeleton,

### FE Simulation of Hand Grasping, Model Validation and Sensitivity Analysis

To validate this FE human hand, the three *in-vivo* grasping tests were simulated. The simulated contact pressure at five fingertips and the contact area across the whole hand were compared to their measured counterparts. The contact pressure was detected by the data glove during the *in-vivo* experiment. Red paint was daubed onto the subject’s hand and paper was wrapped onto the surface of the objects to capture the contact area of the hand. Photos of the handprints were taken with a scale and imported into Creo (PTC, Creo Parametric, US) to measure the area. Each grasp was performed six times to obtain the average values and standard deviations.

A series of sensitivity analyses was conducted to investigate the effect of material properties and muscle forces on contact pressure and contact area. The findings can be applied as guidance to the design of bionic hands in the future, such as in the material selection. To perform the material sensitivity study, the experimental results of the uniaxial tensile test available in the literature^18^,^19^ were modified. The stress-strain curve of the skin and subcutaneous tissue were adjusted by modifying the experimental stress values by ± 5 and ± 10% while the strain values were kept unchanged. The modified stress-strain data was then fitted again and input into the Ogden model by using the ‘material evaluate’ module in Abaqus to obtain the new material properties. Finally, sensitivity analysis was carried out based on these modified material parameters. For illustration, only cylindrical grasping was simulated for sensitivity analysis of material properties.

The forces of FDS, FDP, AP, and FPB muscles were varied by ± 5, ± 10% from their baseline values for the sensitivity study. For each trial, only one muscle force was modified while other forces remained unchanged to study the sensitivity of this particular force. The modified material properties and muscle forces are presented in the Tables S2 to S6, Figs. S1 and S2 in the supplementary material of this paper.

## Results

**Figure 4:**
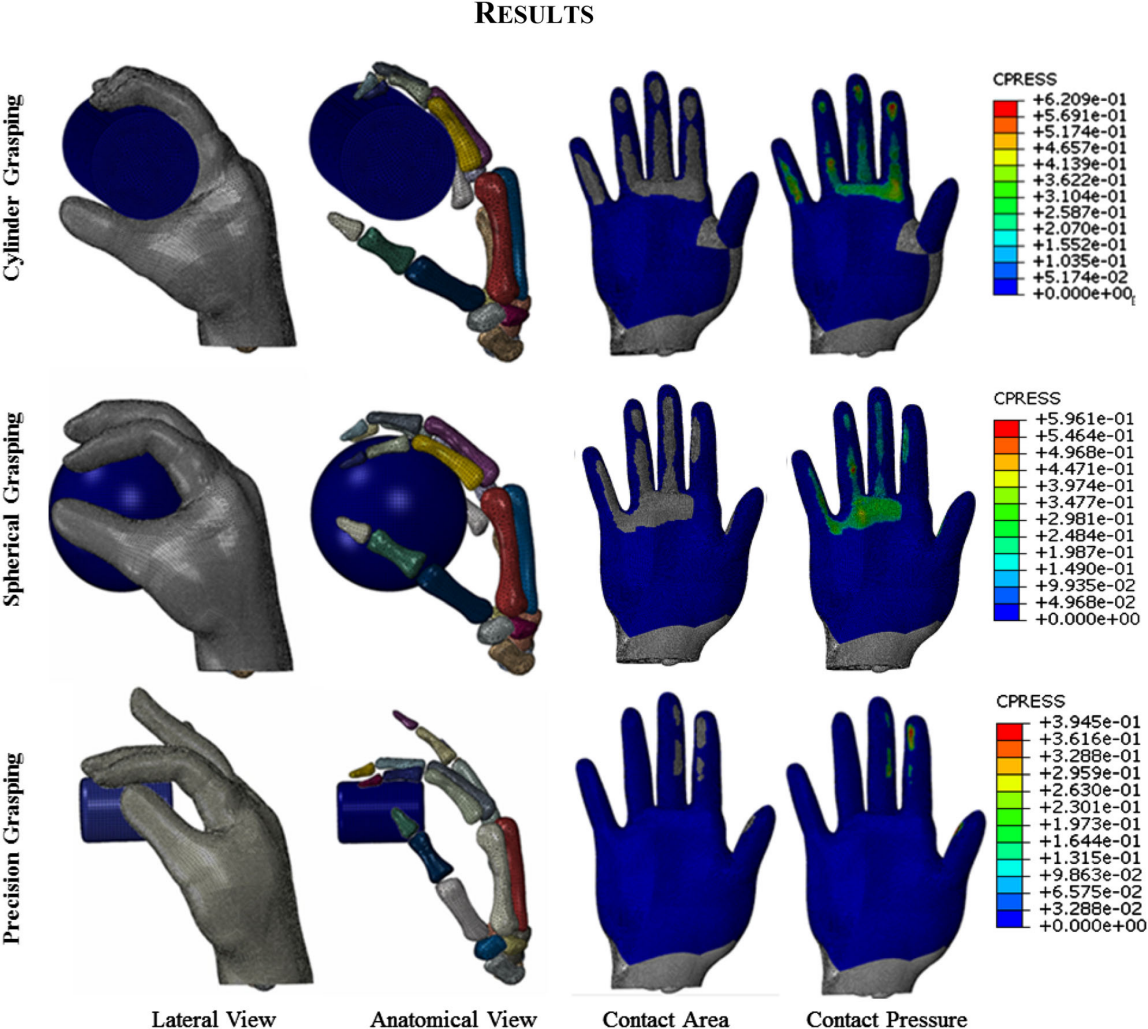
Predicted contact area and pressure during three grasping.

**Figure 5:**
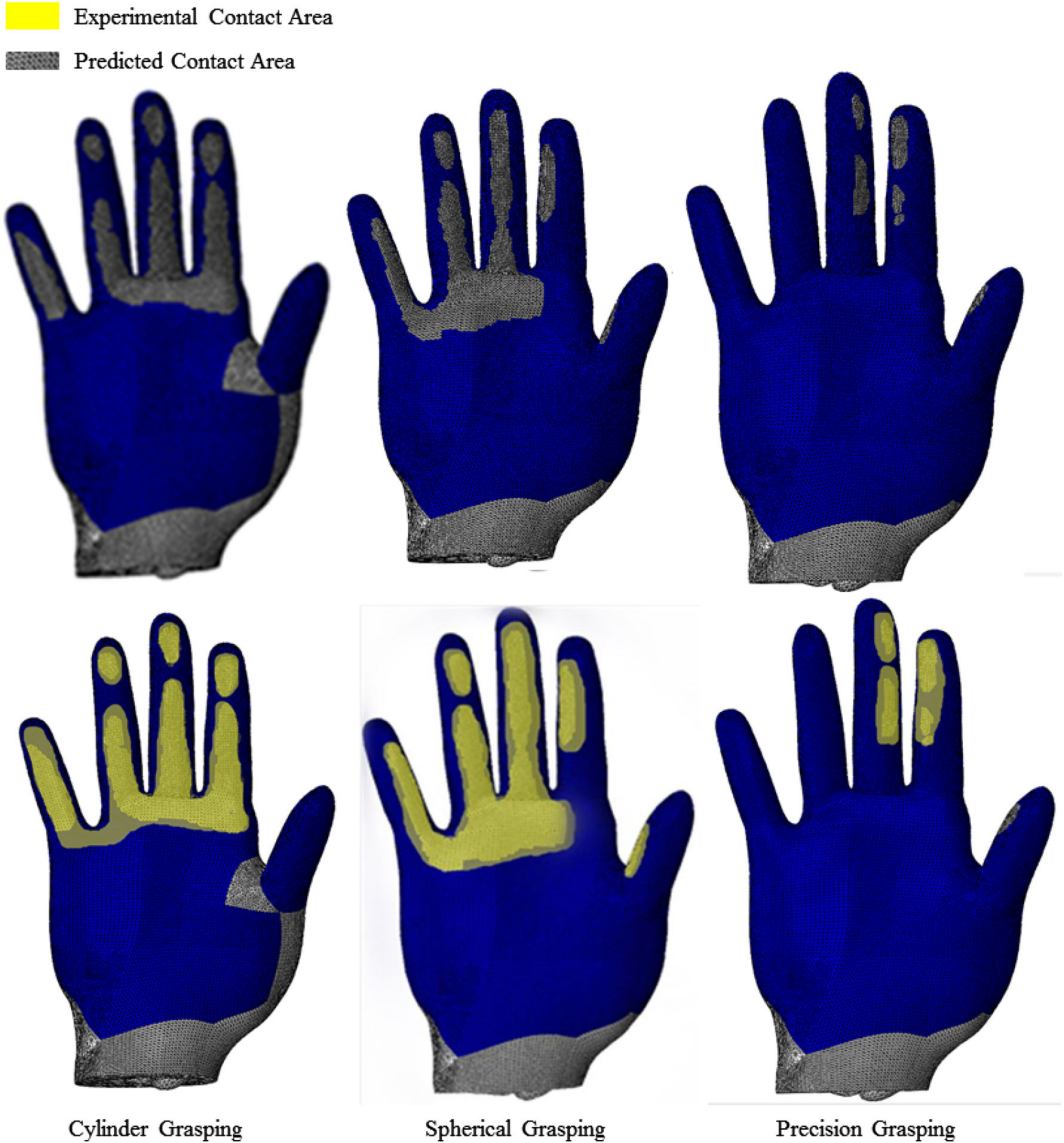
Comparison of contact area between experimental measurement and FE prediction.

**Figure 6:**
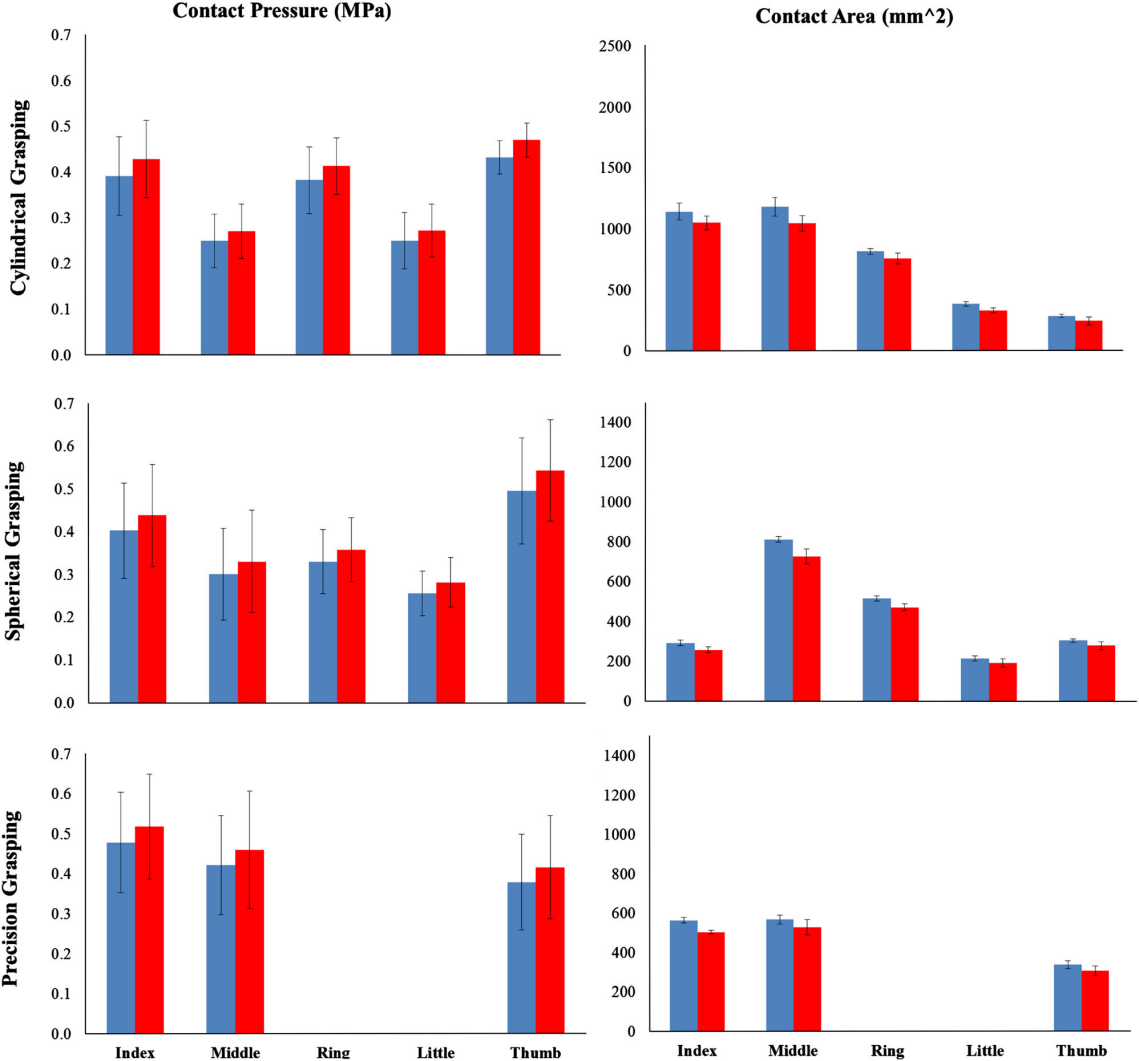
Comparison between the simulated (red) and the *in-vivo* measured (blue) contact pressures on the finger tips and contact area of the whole hand. The experimental and simulation results are shown with standard deviations.

**Figure 7:**
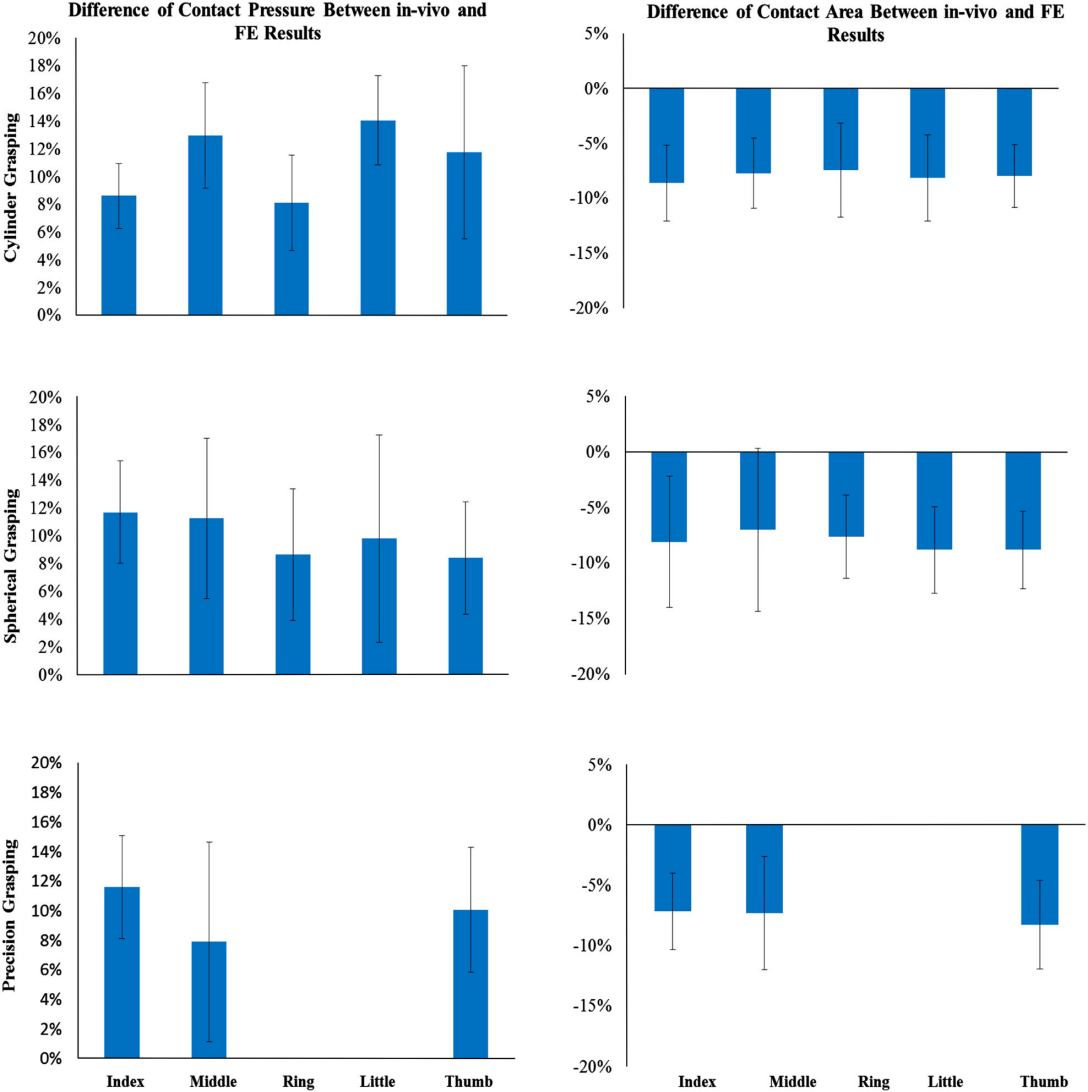
Relative differences between experimental and predicted results.

Figure 4 shows the predicted contact pressure and contact area for the three different grasping and Fig. 5 shows a comparison between the FE predicted and experimental measured contact areas. It is clear that the predicted contact areas matched the *in-vivo* experiment results very well for all three of the grasping. The hand was divided into six different contact regions: five fingers and one palm. Results in each region are presented and compared. As mentioned before, each *in-vivo* grasping test was performed for six times. The muscle forces and joint angles from each test were imported into the FE model, resulting in 6 simulations. The numerical model agreed well with the *in-vivo* experimental results, as shown in Fig. 6. The highest contact pressure observed was on the distal fingertip of the thumb during cylindrical and spherical grasping. In the case of precision grasping, the highest contact pressure appeared on the index finger rather than the other fingers. It can be seen that the contact area varied greatly among six contact regions for each particular grasping posture and also among three different grasping actions. The relative differences between the predicted and experimental results are shown in Fig. 7 together with their standard deviations. The relative differences of contact pressure for the cylindrical and spherical grasping were all below 20% and the differences were all less than 16% for precision grasping. The relative differences for the contact area in all grasps were below 15%. In all cases, the *in-vivo* contact pressure was less than the simulated results. By contrast, all of the contact areas from the *in-vivo* experiments were larger than the simulated values by 7.9 to 14.1%. All of the simulation results lay within the range of deviations of the experimental results. Therefore the difference between experiments and simulations are not statically significant.

Tables 4 and 5 present the effects of skin and subcutaneous tissue material properties on the contact pressure and area. Only cylindrical grasping was selected for material property sensitivity analysis for illustration. Contact pressure on the fingertip increased with the hardened tissue and decreased with softened tissue. The change in contact pressure varied disproportionally with the increasing nominal stress. Both the contact pressure and contact area were more sensitive to variations in skin properties than to variations in subcutaneous tissue properties. The maximum ratio of percentage change for subcutaneous tissue was 2.04 for the contact area and 1.20 for contact pressure, while for skin, it was 1.53 and 0.77 respectively. The hardened subcutaneous tissue (ratio of percentage change ranged from 1.6 to 9.4%) had a more significant effect than softened tissue (0.95 to 5.90%) on contact pressure, while the softened skin (2.7 to 8.0%) caused larger pressure changes than the hardened skin (1.0 to 4.5%). The contact area was more sensitive to the hardened material for both skin and subcutaneous tissue. The ratios of percentage change for both contact pressure and area caused by subcutaneous tissue were well above those of the skin.

**Table 4:** Simulated contact pressure and their percentage changes (%) when the material property was varied from its baseline values.

Contact region	(− 10%)	(− 5%)	(Baseline)	(+ 5%)	(+ 10%)
Change in the nominal stress of the tensile test data of skin in Ref. 16 and 36					
Index	0.478 (− 4.1)	0.4852 (− 2.66)	0.4984	0.5035 (+ 1.02)	0.5084 (+ 2.00)
Middle	0.2195 (− 8.15)	0.2241 (− 6.23)	0.2390	0.2435 (+ 1.89)	0.2497 (+ 4.49)
Ring	0.4487 (− 4.76)	0.4585 (− 2.68)	0.4711	0.478 (+ 1.46)	0.4866 (+ 3.28)
Little	0.2513 (− 7.98)	0.2522 (− 7.66)	0.2731	0.2793 (+ 2.26)	0.2811 (+ 2.92)
Thumb	0.464 (− 4.29)	0.4687 (− 3.32)	0.4848	0.4907 (+ 1.22)	0.498 (+ 2.73)
Change in the nominal stress of the tensile test data of subcutaneous tissue in Ref. 16 and 36					
Index	0.4823 (− 3.24)	0.4907 (− 1.55)	0.4984	0.511 (+ 2.52)	0.5347 (+ 7.27)
Middle	0.2292 (− 4.09)	0.2307 (− 3.46)	0.2390	0.2487 (+ 4.06)	0.2614 (+ 9.38)
Ring	0.458 (− 2.79)	0.4652 (− 1.26)	0.4711	0.4789 (+ 1.65)	0.4838 (+ 2.69)
Little	0.257 (− 5.90)	0.2676 (− 2.02)	0.2731	0.2814 (+ 3.03)	0.2979 (+ 9.07)
Thumb	0.4711 (− 2.82)	0.4802 (− 0.95)	0.4848	0.5139 (+ 6.01)	0.5262 (+ 8.54)

Contact pressure in MPa (Percentage change % w.r.t. baseline)

**Table 5:** Simulated contact area and their percentage changes (%) when the material property was varied from its baseline values.

Contact region	(− 10%)	(− 5%)	(Baseline)	(+ 5%)	(+ 10%)
Change in the nominal stress of the tensile test data of skin in Ref. 16 and 36					
Index	976.90 (− 3.00)	988.54 (− 1.85)	1007.15	1018.93 (+ 1.17)	1033.72 (+ 2.64)
Middle	971.54 (− 2.50)	982.7 (− 1.38)	996.48	1009.40 (+ 1.30)	1021.86 (+ 2.55)
Ring	684.26 (− 3.69)	698.33 (− 1.71)	710.47	722.90 (+ 1.75)	731.48 (+ 2.96)
Little	298.73 (− 7.11)	309.81 (− 3.66)	321.59	333.19 (+ 3.61)	346.28 (+ 7.68)
Thumb	213.77 (− 6.23)	219.69 (− 3.64)	227.98	232.45 (+ 1.96)	241.90 (+ 6.11)
Change in the nominal stress of the tensile test data of subcutaneous tissue in Ref. 16 and 36					
Index	971.25 (− 3.56)	990.44 (− 1.66)	1007.15	1028.97 (+ 2.17)	1045.73 (+ 3.83)
Middle	952.17 (− 4.45)	976.53 (− 2.00)	996.48	1015.68 (+ 1.93)	1038.47 (+ 4.21)
Ring	662.16 (− 6.80)	682.89 (− 3.88)	710.47	738.42 (+ 3.93)	759.6 (+ 6.92)
Little	264.19 (− 17.85)	298.77 (− 7.10)	321.59	356.78 (+ 10.94)	387.14 (+ 20.38)
Thumb	201.70 (− 11.53)	216.38 (− 5.09)	227.98	231.76 (+ 1.66)	247.66 (+ 8.63)

Contact area in mm^2^ (Percentage change % w.r.t. baseline)

Tables 6 and 7 show the effects of varying four main muscle forces on contact pressure and contact area during cylindrical grasping. In general, the predicted average contact pressure of the fingertips varied with the corresponding muscle forces’ trends. The contact area on all the fingers varied non-linearly but still followed the variation trend of muscle forces.

**Table 6:** Simulated contact pressure and their percentage change (%) when the muscle forces were varied from their baseline values.

Contact region	(− 20%)	(− 10%)	(Baseline)	(+ 10%)	(+ 20%)
Percentage change of FDS muscle force from its baseline value (138.78 N)					
Index	0.4778 (− 4.14)	0.4865 (− 2.40)	0.4984	0.5104 (+ 2.40)	0.5197 (+ 4.27)
Middle	0.2258 (− 5.51)	0.228 (− 4.59)	0.2390	0.2597 (+ 8.67)	0.2607 (+ 9.09)
Ring	0.4659 (− 1.11)	0.4697 (− 0.30)	0.4711	0.4805 (+ 1.99)	0.4916 (+ 4.35)
Little	0.2587 (− 5.28)	0.2688 (− 1.58)	0.2731	0.2819 (+ 3.22)	0.2936 (+ 7.50)
Thumb	0.4848 (0.00)	0.4848 (0.00)	0.4848	0.4848 (0.00)	0.4848 (0.00)
Percentage change of AP muscle force from its baseline value (26.68 N)					
Index	0.4876 (− 2.17)	0.4923 (− 1.23)	0.4984	0.5017 (+ 0.65)	0.5096 (+ 2.24)
Middle	0.2308 (− 3.42)	0.2374 (− 0.66)	0.2390	0.2415 (+ 1.06)	0.2498 (+ 4.53)
Ring	0.4711 (0.00)	0.4711 (0.00)	0.4711	0.4711 (0.00)	0.4711 (0.00)
Little	0.2731 (0.00)	0.2731 (0.00)	0.2731	0.2731 (0.00)	0.2731 (0.00)
Thumb	0.425 (− 12.33)	0.4405 (− 9.13)	0.4848	0.5255 (+ 8.40)	0.559 (+ 15.31)

**Table 7:** Simulated contact area and their percentage change (%) when the muscle forces were varied from their baseline values.

Contact region	(− 20%)	(− 10%)	(Baseline)	(+ 10%)	(+ 20%)
Percentage change of FDS muscle force from its baseline value (138.78 N)					
Index	958.59 (− 4.82)	975.79 (− 3.11)	1007.15	1028.74 (+ 2.14)	1037.30 (+ 2.99)
Middle	965.60 (− 3.10)	981.25 (− 1.53)	996.48	1008.28 (+ 1.18)	1026.74 (+ 3.04)
Ring	687.77 (− 3.20)	696.57 (− 1.96)	710.47	725.48 (+ 2.11)	738.90 (+ 4.00)
Little	287.58 (− 10.58)	305.24 (− 5.08)	321.59	339.20 (+ 5.48)	347.27 (+ 7.99)
Thumb	227.98 (0.00)	227.98 (0.00)	227.98	227.98 (0.00)	227.98 (0.00)
Percentage change of FDS muscle force from its baseline value (89.37 N)					
Index	984.18 (− 2.28)	991.25 (− 1.58)	1007.15	1017.50 (+ 1.03)	1034.28 (+ 2.69)
Middle	984.67 (− 1.19)	990.70 (− 0.58)	996.48	1004.25 (+ 0.78)	1019.40 (+ 2.30)
Ring	687.16 (− 3.28)	696.80 (− 1.92)	710.47	721.58 (+ 1.56)	734.16 (+ 3.33)
Little	297.49 (− 7.49)	310.68 (− 3.39)	321.59	330.80 (+ 2.86)	338.47 (+ 5.25)
Thumb	227.98 (0.00)	227.98 (0.00)	227.98	227.98 (0.00)	227.98 (0.00)
Percentage change of FDS muscle force from its baseline value (26.68 N)					
Index	992.24 (− 1.48)	1001.80 (− 0.53)	1007.15	1019.68 (+ 1.24)	1028.47 (+ 2.12)
Middle	984.27 (− 1.23)	990.40 (− 0.61)	996.48	1000.57 (+ 0.41)	1006.54 (+ 1.01)
Ring	710.47 (0.00)	710.47 (0.00)	710.47	710.47 (0.00)	710.47 (0.00)
Little	321.59 (0.00)	321.59 (0.00)	321.59	321.59 (0.00)	321.59 (0.00)
Thumb	208.72 (− 8.45)	220.14 (− 3.44)	227.98	237.17 (+ 4.03)	257.60 (+ 12.99)

Varying the FDS muscle force within the range of ± 20% caused a maximum 9.09% contact pressure variation at middle fingertip during cylindrical grasping. However, the contact pressures on fingertips are not very sensitive to the variation in FDS muscle forces since the contact pressure is limited to vary from 0.30 to 9.1% which is well below the muscle forces variation range of ± 20%. For the effect of FDS force on the contact area, it was found that the contact area on the Little finger was the most sensitive part to muscle force variation with a maximum change of 10.6%.

FDP is the only muscle tendon which is connected to the distal phalanx. It may cause a significant effect on the contact pressure on the fingertips. A 20.0% increase in FDP muscle force raised the contact pressure on the middle fingertip by 23.7% during cylindrical grasping, and by 4.0 to 22.8% on other fingertips, much higher than the 0.3 to 9.1% variations caused by FDS muscle force. The FDS inserts into middle phalanges and therefore has less of an effect on fingertip pressure than the FDP muscle. As for the contact area, a ± 20% change of FDP muscle force only affected the contact area by less than 7.5% for the scenario of cylindrical grasping. All the ratios of percentage change for contact area were below 0.55 while the maximum ratio of percentage change for contact pressure can be up to 1.19 on the middle fingertip.

Although the FDS and FDP muscle forces have little effect on the contact on the distal part of the thumb, this is not the case for AP muscle force. A 20% increase of the AP muscle force increased the contact pressure on the thumb by 15.3%, with the ratio of percentage change being 0.77. The contact pressure and area on the index and middle digits were also affected by AP muscle force due to its insertion into the second and third metacarpal bones. The contact area on the thumb was increased by 13.0% when the AP was increased by 20.0% for cylindrical grasping, equivalent to a percentage change ratio of 0.65. In all cases, the contact pressure and contact area changed nonlinearly with the varying material properties and muscle forces. This reflects the nature of the nonlinear contact problem and the nonlinear behaviour of the skin and soft tissues. The sensitivity analysis results for varying muscle forces during spherical and precision grasping can be found in Tables S7 to S10 in the supplementary material.

## Discussion

The FE model in this study made use of the subject-specific human hand geometry, muscle forces (loading conditions) and joint angles (boundary conditions) which were defined based on the *in-vivo* experiment results of the same subject. By contrast, most of the researchers^7^,^19^ developed their FE hand models based on geometry, loading and kinematics from different subjects. It is also common practice in existing research to regard the skin and subcutaneous tissue as a single part, although the multi-layered structure of the hand is critical for simulating the contact mechanisms. None of the existing models were developed using the detailed and anatomically intact geometry of the human hand. All the existing FE models of the human hand cannot be validated against the corresponding subject-specific *in-vivo* test results.^7^,^8^,^19^ The interphalangeal ligaments and extensor tendon structures were modelled as non-linear spring elements. In total, 68 spring-like elements were employed in this FE human hand, unlike the existing work in which ligaments and tendons were excluded. Moreover, the material properties of the soft tissues were represented by Ogden model which brings the hyper-elastic behaviour of human skin into consideration rather than the simplified isotropic linear elastic material behaviour employed by other researchers.^7^,^8^,^41^

The predicted contact pressure and contact area were in good agreement with the *in-vivo* experiment results for all three grasping. The average relative differences between the predicted and *in-vivo* measured contact pressure on each finger were all below 12.0%, except on the middle and little digit, where differences of 14.1 and 13.0% were recorded during cylindrical grasping respectively. Two main sources may contribute to the difference between the predictions and experimental results. Firstly, the anisotropic viscoelastic material behaviour of the hand was simplified by employing the Ogden model. Secondly, the contact pressure was detected by the data glove during the *in-vivo* grasping test and this data glove was not included in the FE model. The relative differences for the contact area were all below 10% and a larger difference was observed on the digits for cylindrical grasping. Most of the predicted contact areas were smaller than the experimental results. This may be due to the use of daubed paint onto the subject’s hand. It has been reported that a wet hydration condition can significantly affect the skin’s deformation and increase the contact area,^1^,^3^ while this effect was not considered in this FE human hand model. The comparison above demonstrates that the FE hand model developed in this study can simulate hand contact accurately. This detailed and subject-specific FE hand model has the ability to reveal the complex biomechanical properties and contact mechanisms of the human hand.

Sensitivity analyses were carried out to investigate how the material and loading parameters influence the hand contact. The material properties (stiffness) represent material’s ability to resist deformation. It is easier to compress a soft material than a hard material. When contact occurs, soft material would have a larger contact area or lower contact pressure than hard material. The sensitivity analysis did show that the average contact pressures on all distal phalanges decreased with softened skin or subcutaneous tissue and vice versa with hardened material properties. The change of material properties also affected the contact area. Both the contact pressure and area varied nonlinearly with the material properties.

The increased muscle forces led to increased contact pressure and contact area as expected. The contact pressure was affected more intensely by varying FDP muscle force rather than FDS. A 24.0% increase in contact pressure on one of the distal phalanges was produced by a 20.0% increase in FDP muscle forces during cylindrical grasping, but only 9.1% by FDS muscle forces. FDS tendons were inserted onto the middle phalanges and FDP was the only tendon which inserted onto the distal phalanges. Therefore the effect of varying FDP muscle should have a larger impact on fingertip contact pressure than FDS muscle. In most of the scenarios, increasing muscle forces can cause larger increments on contact pressure and all of the contact pressure increments were less than the corresponding muscle force increments. These can be caused by the Ogden material model applied to soft tissues and the contact property defined in this FE model. In contrast to the contact pressure, the FDP tendon force had a more significant effect on the contact area than the FDP since the MVC of FDS was larger than that of the FDP.

The sensitivity analysis results of muscle forces during spherical and precision grasping were similar to those during cylindrical grasping and are listed in the supplementary material. The material properties were properly defined in this FE hand model since the predictions were close to the *in-vivo* experimental results. Furthermore, the quasi-static FE simulations of the entire grasping were conducted in only one single step, which means that the metacarpophalangeal, proximal interphalangeal, and distal interphalangeal joints were rotated simultaneously, and so the fully tendon driven system can provide a better prediction of the contact mechanics. Very few of the existing FE simulations had been done on full hand contact problems due to high dexterity, multiple degrees of freedom of the human hand, high computational demands and complicated interactions between tissue layers or objects.

Although this finite element modelling is subject-specific, the approach/technique can be employed to build FE model for different human hands. In a certain level, the sensitivity analysis conducted is equivalent to looking at what a different subject would like and the findings are general features. More work is required to investigate the effect of subject morphologies in the future and this involves a huge amount of repetitive work to develop FE models from DICOM data of different subjects.

To the best of the authors’ knowledge, this FE human hand model is the first validated computational model based on subject-specific data. It can provide a powerful numerical tool to study biomechanics or even neuro-related human perception mechanisms.^10^,^14^ Some basic biomechanical problems such as the complicated topology of the extensor tendon can be investigated by using this FE model. Although some very simple numerical models of tissue specimens have already been developed, they cannot study the whole process of human sensory-motor control.^15^,^38^,^39^,^47^ This FE hand model has the potential to provide real-time neural spike information during active touch and other manipulation processes. The mechanical parameter which is related to human tactile perception such as strain energy density at the locations of mechanoreceptors can be derived to predict the neural signal.^10^,^23^,^42^ The adjusted neural activation level of muscle synergy modes can also be implemented in the FE model as feedback results after decoding the perceived information.^4^,^21^ The existing research has revealed that similar neural dynamics of the 1st order tactile neuron were captured from different subjects.^13^,^23^ Therefore, the present FE human hand model can be employed for the neurophysiological investigation although it is subject-specific. The morphology of hand or size of muscle can affect the grasping mechanisms while the size of hand or the maximum voluntary contraction muscle forces of different subjects can be scaled based on our subject-specific hand model. Different bionic hands can be designed after the scaling of MVCs and morphology. Similar scaling methods have been used by other researchers and have been applied for the investigations on biomechanical aspect.^17^,^34^ Therefore the deep understanding of how the human hand perceives or manipulates objects can be achieved based on this FE hand model and the findings can also be applied onto biologically inspired robotic hands or neuro-robotic hand designs.

A subject-specific FE human hand model was developed. A new method was proposed to define the loading and boundary conditions based on the *in-vivo* test results of the same subject. Validation against the *in-vivo* test results demonstrated that this model can provide accurate predictions on human hand contact pressure and contact pattern. The sensitivity analyses showed that the contact area and contact were moderately sensitive to material properties and significantly sensitive to loading conditions. This indicates that material selection and contraction capabilities of artificial muscles for future bionic hand designs are crucial for mimicking the biomechanical properties of a real human hand. This study proved that FE simulation is a practical method to investigate human perception and manipulation based on biomechanical and neurophysiological aspects.

## Electronic supplementary material

Below is the link to the electronic supplementary material.

Supplementary material 1 (PDF 571 kb)
