# Supplementary material for: Subject-Specific Finite Element Modelling of the Human Hand Complex: Muscle-Driven Simulations and Experimental Validation
Source: Ann Biomed Eng. 2019 Dec 16;48(4):1181–95. doi: 10.1007/s10439-019-02439-2 (PMC7089907; doi:10.1007/s10439-019-02439-2)
Supplement: Supplementary file 1 — Supplementary material 1 (PDF 571 kb) [file 10439_2019_2439_MOESM1_ESM.pdf]

Supplementary Material for “Subject-Specific Finite Element Modelling of the Human Hand Complex: Muscle-Driven Simulations and Experimental Validation”

Yuyang Wei, Zhenmin Zou, Guowu Wei, Zhihui Qian* and Lei Ren*

Annals of Biomedical Engineering

In this supplementary file, we report the material properties of skin and subcutaneous tissues for sensitivity analysis and also the simulation results of spherical and precision grasping for the sensitivity analysis.

The sensitivity analyses were conducted to investigate the effect of material properties on the simulation results. The published tensile test results were modified, the stress-strain curve of skin and subcutaneous tissue were adjusted by modifying the experimental stress by $\pm$5% and $\pm$10% while the corresponding strain values were kept unchanged. The modified stress-strain data were then fitted again and input into the Ogden model by using the ‘material evaluate’ module of Abaqus to obtain the new material properties. The sensitivity analysis was carried out based on these modified material parameters.

Table S1 presents the stiffness of the spring elements for mimicking the ligaments.

Figure S1 and S2 present the fitted strain-stress curve for the modified material properties of skin and subcutaneous tissue respectively.

Table S2 and S3 present the modified material properties of skin and subcutaneous tissue obtained from the modified tensile test results respectively. These parameters define the adjusted hyperelastic material properties for sensitivity analysis.

Table S4 to S7 present the varied muscle forces of three different grasping for sensitivity analysis.

Table S7 and S8 show the simulated contact pressure and their percentage changes when the muscle forces are changed from their baseline values during spherical and precision grasping for sensitivity analysis respectively.

Table S9 and S10 show the simulated contact area and their percentage changes when the muscle forces are changed from their baseline values during spherical and precision grasping for sensitivity analysis respectively.

TABLE S1

LIST OF LIGAMENTS MODELED SPECIFYING THE CONNECTIONS AND STIFFNESS

Ligament	Connection 1	Connection 1	Stiffness (N/mm)
Collateral ligament	Phalange	Phalange	71
Volar plate 1	Distal Phalange	Medial Phalange	23
Volar plate 2	Medial Phalange	Proximal phalange	22
Volar plate 3	Proximal phalange	Metacarpal bone	74
Deep transverse metatarsal ligament	Phalange	Phalange	4
Dorsal intercarpal	Hamate	Capitate	325
Dorsal intercarpal	Capitate	Trapezoid	300
Dorsal intercarpal	Hamate	Triquetrum	300
Dorsal intercarpal	Hamate	Lunate	150
Dorsal intercarpal	Capitate	Lunate	150
Dorsal intercarpal	Capitate	Scaphoid	150
Dorsal intercarpal	Scaphoid	Trapezium	150
Dorsal lunotriquetral	Lunate	Triquetrum	350
Dorsal scapholunate	Lunate	Scaphoid	230
Radial collateral carpal	Radius	Scaphoid	50
Capitotrapezial	Capitate	Trapezium	300
Capitohamate	Capitate	Hamate	325
Volar triquetrohamate	Hamate	Triquetrum	50
Scaphotrapezial	Scaphoid	Trapezium	150
Radial arcuate	Capitate	Scaphoid	40
Ulnar arcuate	Capitate	Triquetrum	40
Volar scapholunate	Lunate	Scaphoid	230
Volar lunotriquetral	Lunate	Triquetrum	350
Pisohamate	Hamate	Pisiform	100
Radioscaphocapitate	Radius	Capitate	50
Pisotriquetral	Pisiform	Triquetrum	150
Long radiolunate	Lunate	Radius	40
Short radiolunate	Lunate	Radius	50

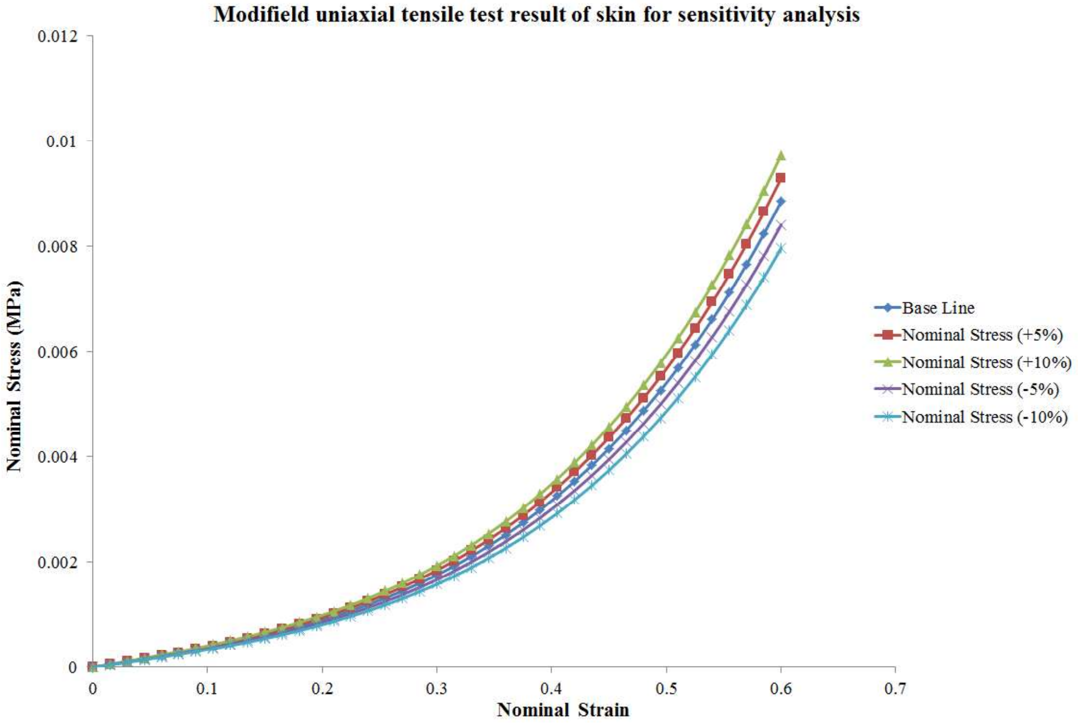

Fig. S1. Modified and fitted tensile test results on skin

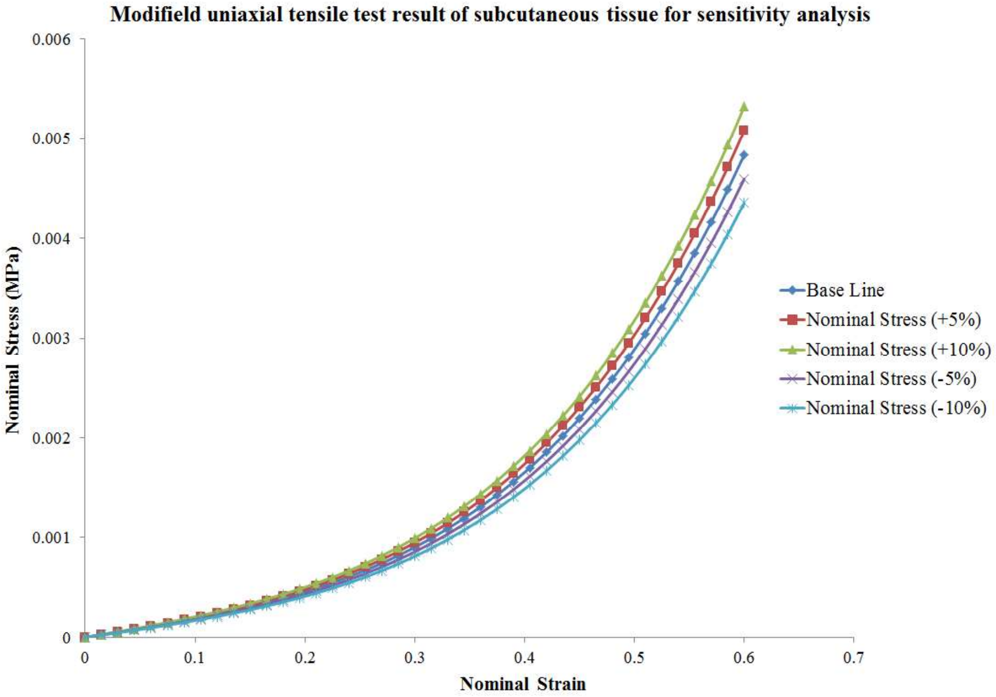

Fig. S2. Modified and fitted tensile test results on subcutaneous tissue

TABLE S2

THE MODIFIED MATERIAL PROPERTY OF SKIN FOR SENSITIVITY ANALYSIS

	Change in the nominal stress of the tensile test data of skin				
Parameters	-10%	-5%	(Base Line)	5%	10%
μ1 (MPa)	-0.0169	-0.0162	-0.07594	-0.0169	-0.0177
α1	5.9335	5.9522	4.9410	6.1833	6.1852
μ2 (MPa)	0.0162	0.11111	0.01138	0.0162	0.0170
α2	6.0460	6.0654	6.4250	6.6332	6.6333
μ3 (MPa)	0.0019	0.00613	0.06572	0.0019	0.0020
α3	4.0752	4.0053	4.7120	2.4846	2.4841

TABLE S3

THE MODIFIED MATERIAL PROPERTY OF SUBCUTANEOUS TISSUE FOR SENSITIVITY ANALYSIS

	Change in the nominal stress of the tensile test data of subcutaneous tissue				
Parameters	-10%	-5%	(Base Line)	5%	10%
μ1 (MPa)	-0.0083	-0.0088	-0.04895	-0.0100	-0.0101
α1	6.5357	6.5353	5.511	6.5357	6.5354
μ2 (MPa)	0.0078	0.0083	0.00989	0.0092	0.0096
α2	6.9511	6.9508	6.571	6.9510	6.9509
μ3 (MPa)	0.0010	0.0010	0.03964	0.0011	0.0012
α3	3.1654	3.1667	5.262	3.1653	3.1663

TABLE S4

THE MODIFIED MUSCLE FORCES FOR SENSITIVITY ANALYSIS DURING CYLINDRICAL GRASPING

	Percentage change of muscle forces from their baseline values				
Muscle	-10%	-5%	Base Line	5%	10%
FDS	124.9N	131.84N	138.78N	145.72N	152.66N
FDP	80.43N	84.9N	89.37N	93.84N	98.31N
AP	24.01N	25.35N	26.68N	28.01N	29.35N

TABLE S5

THE MODIFIED MUSCLE FORCES FOR SENSITIVITY ANALYSIS DURING SPHERICAL GRASPING

	Percentage change of muscle forces from their baseline values				
Muscle	-10%	-5%	Base Line	5%	10%
FDS	216.03N	228.03N	240.03N	252.03N	264.03N
FDP	140.26N	148.05N	155.84N	163.63N	171.42N
FPB	51.3N	54.15N	57N	59.85N	62.7N

TABLE S6

THE MODIFIED MUSCLE FORCES FOR SENSITIVITY ANALYSIS DURING PRECISION GRASPING

	Percentage change of muscle forces from their baseline values				
Muscle	-10%	-5%	Base Line	5%	10%
FDS	100.55N	106.13N	111.72N	117.31N	122.89N
FDP	145.98N	154.09N	162.2N	170.31N	178.42N
FPB	92.34N	97.47N	102.6N	107.73N	112.86N

TABLE S7

SIMULATED CONTACT PRESSURE AND THEIR PERCENTAGE CHANGES (%) WHEN THE MUSCLE FORCES WERE VARIED FORM THEIR BASELINE VALUES (SPHERICAL GRASPING)

Contact Region	Percentage change of FDS muscle force from its baseline value				
	192.02 N (-20%)	216.03 N (-10%)	240.03 N (Baseline)	264.03 N (+10%)	288.04 N (+20%)
Index	0.5218 (-3.26)	0.5320 (-1.37)	0.5394	0.5465 (+1.31)	0.5507 (+2.09)
Middle	0.4027 (-3.31)	0.4113 (-1.25)	0.4165	0.4206 (+0.98)	0.4280 (+2.76)
Ring	0.3654 (-2.70)	0.3701 (-1.45)	0.3755	0.3804 (+1.30)	0.3873 (+3.13)
Pinky	0.2457 (-5.30)	0.2517 (-2.99)	0.2595	0.2638 (+1.67)	0.2697 (+3.95)
Thumb	0.6760 (0.00)	0.6760 (0.00)	0.6760	0.6760 (0.00)	0.6760 (0.00)

Contact Region	Percentage change of FDP muscle force from its baseline value				
	124.68 N (-20%)	140.26 N (-10%)	155.84 N (Baseline)	171.42 N (+10%)	187.01 N (+20%)
Index	0.4970 (-7.86)	0.5213 (-3.36)	0.5394	0.5604 (+3.89)	0.5899 (+9.36)
Middle	0.3740 (-10.20)	0.3927 (-5.72)	0.4165	0.4394 (+5.50)	0.4613 (+10.76)
Ring	0.3329 (-11.35)	0.3541 (-5.71)	0.3755	0.3988 (+6.19)	0.4219 (+12.35)
Pinky	0.2174 (-16.21)	0.2360 (-9.04)	0.2595	0.2883 (+11.11)	0.3111 (+19.90)
Thumb	0.6760 (0.00)	0.6760 (0.00)	0.6760	0.6760 (0.00)	0.6760 (0.00)

Contact Region	Percentage change of FPB muscle force from its baseline value				
	45.6 N (-20%)	51.3 N (-10%)	57 N (Baseline)	62.7 N (+10%)	68.4 N (+20%)
Index	0.5394 (0.00)	0.5394 (0.00)	0.5394	0.5394 (0.00)	0.5394 (0.00)
Middle	0.4165 (0.00)	0.4165 (0.00)	0.4165	0.4165 (0.00)	0.4165 (0.00)
Ring	0.3755 (0.00)	0.3755 (0.00)	0.3755	0.3755 (0.00)	0.3755 (0.00)
Pinky	0.2595 (0.00)	0.2595 (0.00)	0.2595	0.2595 (0.00)	0.2595 (0.00)
Thumb	0.5580 (-17.45)	0.6297 (-6.84)	0.6760	0.7325 (+8.36)	0.8060 (+19.24)

Note: Contact pressure in MPa (Percentage change % w.r.t. baseline)

TABLE S8

SIMULATED CONTACT PRESSURE AND THEIR PERCENTAGE CHANGES (%) WHEN THE MUSCLE FORCES WERE VARIED FORM THEIR BASELINE VALUES (PRECISION GRASPING)

Contact Region	Percentage change of FDS muscle force from its baseline value				
	89.38 N (-20%)	100.55N (-10%)	111.72 N (Baseline)	122.89 N (+10%)	134.06 N (+20%)
Index	0.5697 (-5.19)	0.5778 (-3.84)	0.6009	0.6278 (+4.48)	0.6391 (+6.36)
Middle	0.513 (-7.24)	0.5316 (-3.88)	0.5531	0.5787 (+4.64)	0.5914 (+6.93)
Ring	0	0	0	0	0
Pinky	0	0	0	0	0
Thumb	0.5189 (0.00)	0.5189 (0.00)	0.5189	0.5189 (0.00)	0.5189 (0.00)

Contact Region	Percentage change of FDP muscle force from its baseline value				
	129.76 N (-20%)	145.98 (-10%)	162.2 N (Baseline)	178.42 N (+10%)	194.64 N (+20%)
Index	0.5219 (-13.14)	0.5604 (-6.73)	0.6009	0.6484 (+7.91)	0.6913 (+15.05)
Middle	0.4764 (-13.86)	0.5137 (-7.12)	0.5531	0.5987 (+8.25)	0.6420 (+16.08)
Ring	0	0	0	0	0
Pinky	0	0	0	0	0
Thumb	0.5189 (0.00)	0.5189 (0.00)	0.5189	0.5189 (0.00)	0.5189 (0.00)

Contact Region	Percentage change of FPB muscle force from its baseline value				
	82.08 N (-20%)	92.34 N (-10%)	102.6 N (Baseline)	112.86 N (+10%)	123.12 N (+20%)
Index	0.6009 (0.00)	0.6009 (0.00)	0.6009	0.6009 (0.00)	0.6009 (0.00)
Middle	0.5531 (0.00)	0.5531 (0.00)	0.5531	0.5531 (0.00)	0.5531 (0.00)
Ring	0	0	0	0	0
Pinky	0	0	0	0	0
Thumb	0.4590 (-11.55)	0.4812 (-7.27)	0.5189	0.5630 (+8.49)	0.6012 (+15.86)

Note: Contact pressure in MPa (Percentage change % w.r.t. baseline)

TABLE S9

SIMULATED CONTACT AREA AND THEIR PERCENTAGE CHANGES (%) WHEN THE MUSCLE FORCES WERE VARIED FORM THEIR BASELINE VALUES (SPHERICAL GRASPING)

Contact Region	Percentage change of FDS muscle force from its baseline value				
	192.02 N (-20%)	216.03 (-10%)	240.03 N (Baseline)	264.03 N (+10%)	288.04 N (+20%)
Index	234.15 (-7.00)	243.70 (-3.21)	251.77	269.16 (+6.91)	275.74 (+9.52)
Middle	711.50 (-5.53)	743.54 (-1.27)	753.14	792.90 (+5.28)	814.86 (+8.20)
Ring	450.40 (-6.03)	468.13 (-2.33)	479.28	497.69 (+3.84)	516.24 (+7.71)
Pinky	161.27 (-13.89)	169.85 (-9.31)	187.29	196.24 (+4.78)	208.70 (+11.43)
Thumb	277.41 (0.00)	277.41 (0.00)	277.41	277.41 (0.00)	277.41 (0.00)

Contact Region	Percentage change of FDP muscle force from its baseline value				
	124.68 N (-20%)	140.26 N (-10%)	155.84 N (Baseline)	171.42 N (+10%)	187.01 N (+20%)
Index	235.67 (-6.39)	240.13 (-4.62)	251.77	264.80 (+5.18)	271.39 (+7.79)
Middle	730.86 (-2.96)	742.58 (-1.40)	753.14	769.42 (+2.16)	778.64 (+3.39)
Ring	459.61 (-4.10)	476.52 (-0.58)	479.28	491.63 (+2.58)	502.90 (+4.93)
Pinky	172.33 (-7.99)	180.27 (-3.75)	187.29	196.74 (+5.05)	201.81 (+7.75)
Thumb	277.41 (0.00)	277.41 (0.00)	277.41	277.41 (0.00)	277.41 (0.00)

Contact Region	Percentage change of FPB muscle force from its baseline value				
	45.6 N (-20%)	51.3N (-10%)	57 N (Baseline)	62.7 N (+10%)	68.4 N (+20%)
Index	251.77 (0.00)	251.77 (0.00)	251.77	251.77 (0.00)	251.77 (0.00)
Middle	753.14 (0.00)	753.14 (0.00)	753.14	753.14 (0.00)	753.14 (0.00)
Ring	479.28 (0.00)	479.28 (0.00)	479.28	479.28 (0.00)	479.28 (0.00)
Pinky	187.29 (0.00)	187.29 (0.00)	187.29	187.29 (0.00)	187.29 (0.00)
Thumb	258.64 (6.77)	267.41 (3.60)	277.41	286.24 (3.18)	297.80 (7.35)

Note: Contact area in mm^2 *(Percentage change % w.r.t. baseline)*

TABLE S10

SIMULATED CONTACT AREA AND THEIR PERCENTAGE CHANGES (%) WHEN THE MUSCLE FORCES WERE VARIED FORM THEIR BASELINE VALUES (PRECISION GRASPING)

Contact Region	Percentage change of FDS muscle force from its baseline value				
	89.38N (-20%)	100.55N (-10%)	111.72N (Baseline)	122.89N (+10%)	134.06N (+20%)
Index	494.17 (-4.72)	504.26 (-2.77)	518.65	528.16 (+1.83)	537.48 (+3.63)
Middle	472.67 (-3.96)	486.50 (-1.15)	492.16	501.67 (+1.93)	514.17 (+4.47)
Ring	0	0	0	0	0
Pinky	0	0	0	0	0
Thumb	284.77 (0.00)	284.77 (0.00)	284.77	284.77 (0.00)	284.77 (0.00)

Contact Region	Percentage change of FDP muscle force from its baseline value				
	129.76N (-20%)	145.98N (-10%)	162.2N (Baseline)	178.42N (+10%)	194.64N (+20%)
Index	501.47 (-3.31)	512.68 (-1.15)	518.65	527.40 (+1.69)	539.77 (+4.07)
Middle	477.60 (-2.96)	483.94 (-1.67)	492.16	503.60 (+2.32)	518.45 (+5.34)
Ring	0	0	0	0	0
Pinky	0	0	0	0	0
Thumb	284.77 (0.00)	284.77 (0.00)	284.77	284.77 (0.00)	284.77 (0.00)

Contact Region	Percentage change of FPB muscle force from its baseline value				
	82.08N (-20%)	92.34N (-10%)	102.6N (Baseline)	112.86N (+10%)	123.12N (+20%)
Index	518.65 (0.00)	518.65 (0.00)	518.65	518.65 (0.00)	518.65 (0.00)
Middle	492.16 (0.00)	492.16 (0.00)	492.16	492.16 (0.00)	492.16 (0.00)
Ring	0	0	0	0	0
Pinky	0	0	0	0	0
Thumb	265.41 (-6.80)	271.40 (-4.70)	284.77	297.41 (+4.44)	310.50 (+9.04)

Note: Note: Contact area in mm^2 (Percentage change % w.r.t. baseline)
